# Generation of functional hippocampal neurons from self-organizing human embryonic stem cell-derived dorsomedial telencephalic tissue

**DOI:** 10.1038/ncomms9896

**Published:** 2015-11-17

**Authors:** Hideya Sakaguchi, Taisuke Kadoshima, Mika Soen, Nobuhiro Narii, Yoshihito Ishida, Masatoshi Ohgushi, Jun Takahashi, Mototsugu Eiraku, Yoshiki Sasai

**Affiliations:** 1 Laboratory for Organogenesis and Neurogenesis, RIKEN Center for Developmental Biology, 2-2-3 Minatojima-Minamimachi, Chuo-ku, Kobe 650-0047, Japan; 2 Laboratory for in vitro Histogenesis, RIKEN Center for Developmental Biology, 2-2-3 Minatojima-Minamimachi, Chuo-ku, Kobe 650-0047, Japan; 3 Department of Clinical Application, Center for iPS Cell Research and Application, Kyoto University, 53 Kawahara-cho, Shogoin, Sakyo-ku, Kyoto 606-8507, Japan

## Abstract

The developing dorsomedial telencephalon includes the medial pallium, which goes on to form the hippocampus. Generating a reliable source of human hippocampal tissue is an important step for cell-based research into hippocampus-related diseases. Here we show the generation of functional hippocampal granule- and pyramidal-like neurons from self-organizing dorsomedial telencephalic tissue using human embryonic stem cells (hESCs). First, we develop a hESC culture method that utilizes bone morphogenetic protein (BMP) and Wnt signalling to induce choroid plexus, the most dorsomedial portion of the telencephalon. Then, we find that titrating BMP and Wnt exposure allowed the self-organization of medial pallium tissues. Following long-term dissociation culture, these dorsomedial telencephalic tissues give rise to Zbtb20^+^/Prox1^+^ granule neurons and Zbtb20^+^/KA1^+^ pyramidal neurons, both of which were electrically functional with network formation. Thus, we have developed an *in vitro* model that recapitulates human hippocampus development, allowing the generation of functional hippocampal granule- and pyramidal-like neurons.

## Abstract (web summary)

*In vitro* differentiation of human pluripotent stem cells (hPSCs) has enabled the generation of neuroectodermal tissues. Here, Sakaguchi *et al.* use a modified neocortical induction method to generate functional hippocampal granule and pyramidal-like neurons, as well as dorsomedial telencephalic tissues from hPSCs.

## Graphical abstract

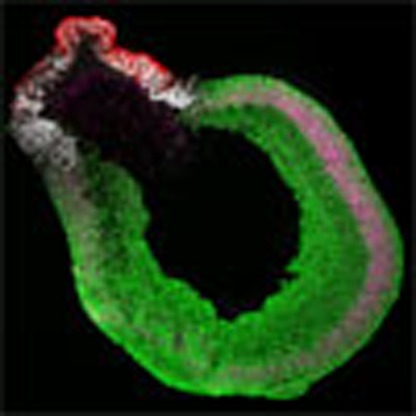

The telencephalon is a well-developed brain region in mammals and functions as a higher integrative centre for various neural information. The telencephalon consists of the neocortex (dorsal pallium) and the limbic cortical systems such as the medial, lateral and ventral pallia, as well as portions of the cerebral nuclei including the amygdala and septum^1^. During development, it is the medial pallium that generates the hippocampus, the brain region known to play a critical role in learning and memory^2^. The medial pallium lies between the neocortex and midline structures such as the roof plate, choroid plexus and cortical hem, and it forms the dorsocaudal portion of the dorsal telencephalon^3^. Hippocampal neurons are composed of granule and pyramidal neurons^2^. Although hippocampal field specification is not complete until a few weeks after birth (in mice), hippocampal field patterning is already specified in the embryonic stage, and presumptive cornu ammonis (CA) fields are able to develop mature pyramidal neurons autonomously^2^. Despite great interest in this area, the mechanisms that govern hippocampal development have remained largely unknown, due in part to a lack of experimentally tractable models for the study of hippocampal development.

In the dorsal-caudal midline, the choroid plexus is the most dorsomedial derivative in the telencephalon^4^, and further lateral to the choroid plexus is the cortical hem. The cortical hem functions as a signalling centre that secretes patterning molecules such as several types of Wnt and bone morphogenetic protein (BMP), and it plays an important role in hippocampal development^5^. On the other hand, the dorsal midline, the future site of the choroid plexus, is a main source for BMP signals^6^. Together, these two signalling centres function as a dorsalizing organizer and are essential to medial pallium patterning.

Over the past several years, substantial progress in human pluripotent stem cell (hPSC) technology has enabled the generation of neuroectodermal tissues *in vitro*^7^,^8^,^9^,^10^,^11^,^12^,^13^. For example, we previously described an efficient three-dimensional (3D) culture method for generating stratified neocortical structure from human embryonic stem cells (hESCs)^11^,^12^. In this method, called serum-free floating culture of embryoid body-like aggregates with quick reaggregation (SFEBq), several thousand dissociated hESCs are quickly reaggregated by plating into each well of a 96-well plate with a special low-adhesion coating. When the floating cell aggregates are cultured in medium suitable for cortical differentiation (Fig. 1a), the majority of aggregates express the telencephalic marker Foxg1 (ref. 12). However, generation of medial pallium from PSCs of any species has not yet been achieved.

In a previous report, we noted that the cortical neuroepithelium (NE) has an internal asymmetry along the rostral-caudal axis; one side preferentially expresses a rostral (frontal) cortical marker, while the other side has stronger expression of a caudal (occipital) cortical marker^12^. These observations show that hESC-derived cortical NE has a self-organizing ability to create a rostral-caudal polarity, suggesting that further refinement of this protocol might facilitate the generation of a medial pallium region.

We therefore sought to modify the previous neocortex induction method to generate medial pallium and hippocampal tissues in 3D culture of hESCs. To this end, we first explored a suitable culture condition for the dominant induction of choroid plexus tissues. We then developed a culture protocol for the partial induction of choroid plexus tissue by transient dorsalizing manipulation. With this approach, we succeeded in generating a continuous structure consisting of choroid plexus, cortical hem and medial pallium tissues. Furthermore, via long-term dissociation culture of the medial pallium tissues, we observed the emergence of mature neurons positive for Prox1 (hippocampal granule neuron marker) and KA1 (CA3 pyramidal neuron marker). These observations indicate that our method generates dorsomedial telencephalic tissues, as well as hippocampal granule- and pyramidal-like neurons from hESCs.

## Results

### Efficient generation of choroid plexus tissues from hESCs

**Figure 1:**
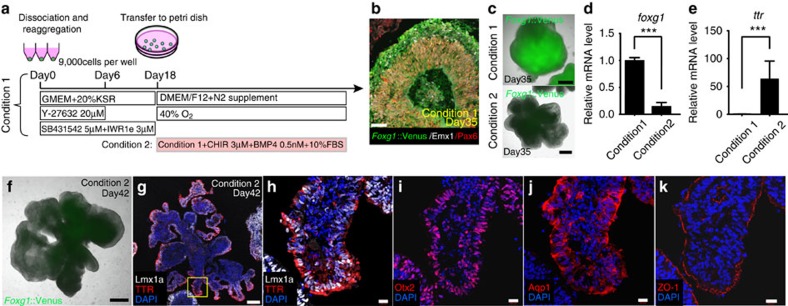
Generation of choroid plexus-like tissues with pleated structure from hESCs. (**a**) Schematic of examined conditions to induce choroid plexus tissues. (**b**) Immunostaining of neocortical structure induced from hESCs on day 35. (**c**) Comparison of aggregate formation from *Foxg1*::Venus knock-in hESCs on day 35. CHIR (GSK3 inhibitor) plus BMP4 treatment (condition 2) induced thin epithelia with many folds with significant attenuation of *Foxg1::*Venus expression. (**d**,**e**) qPCR for genes expressed in dorsomedial telencephalon (***P<0.001). (**d**) *foxg1* significantly attenuated in condition 2 (*n*=3, unpaired *t*-test). (**e**) *ttr* significantly increased in condition 2 (*n*=3, unpaired *t*-test with Welch's correction). (**f**) Bright-field view of one aggregate cultured in condition 2 on day 42. (**g**–**k**) The induction of choroid plexus tissues with pleated structure from hESCs. The pleated epithelia are Lmx1a^+^ (**g**,**h**), Otx2^+^ (**i**) and TTR^+^ (**h**). TTR mainly stained apically (**h**), and Aquaporin-1 stained mainly in apical parts, with less staining in basal parts (**j**). ZO-1 stained the surface of epithelia (**k**). Scale bars, 50 μm (**b**; 500 μm (**c**, **f**); 200 μm (**g**); 20 μm (**h**–**k**). Bars in graph, s.e.m. Nuclear counter staining (blue), 4,6-diamidino-2-phenylindole (DAPI).

The medial pallium lies between the neocortex and the dorsal midline structures including the choroid plexus and cortical hem (Supplementary Fig. 1a). Following a previously described neocortex induction method (Fig. 1a, condition 1)^12^, *Foxg1*::Venus^+^/Emx1^+^/Pax6^+^ cortical NE was reproducibly induced at culture day 35 (Fig. 1b). We hypothesized that the cortical NE could be directed to form medial pallium via a dorsalizing culture environment. We took a two-step approach to accomplish this: first, we defined the culture condition for the dominant differentiation into the most dorsal region (choroid plexus), and then we sought to optimize these dorsalizing effects to induce intermediate region tissues, cortical hem and medial pallium.

To differentiate the hESC-derived cortical NE into dorsomedial tissues, we examined the effects of two factors, Wnt and BMP, both of which are known to promote dorsalization of neural tissues^2^,^11^,^14^. Wnts (Wnt 2b, 3a and 5a) are mainly expressed in cortical hem^5^, and BMPs are apparent in the dorsal midline of the telencephalon including cortical hem (BMP 6 and 7)^5^ and choroid plexus (BMP 2, 4, 5, 6 and 7)^6^. On the basis of these facts, we tested a treatment with BMP4 ligand plus CHIR 99021 (GSK3 inhibitor, also known as Wnt agonist)^10^, a condition designed to mimic the *in vivo* dorsal midline and cortical hem signalling centre (Fig. 1a, condition 2).

Because Foxg1 is not expressed in choroid plexus, we utilized *Foxg1*::Venus knock-in hESCs to monitor the dorsalizing effect in hESC-derived aggregates^12^. After treatment of hESC-derived cortical NE with CHIR+BMP4 (day 18, condition 2), thin epithelia developed in most of the aggregates, and the *Foxg1::*Venus^+^ region almost disappeared (Fig. 1c, condition 2). Consistent with these results, a significant decrease of *foxg1* messenger RNA (mRNA) expression in condition 2 was confirmed using quantitative PCR with reverse transcription (RT–qPCR; Fig. 1d). In addition, *lmx1a* and *otx2*, markers for both cortical hem and choroid plexus, were significantly upregulated in condition 2 compared with condition 1 (Supplementary Fig. 1b,c). Importantly, a choroid plexus-specific marker in telencephalon, transthyretin (*ttr*), was also strongly upregulated in condition 2 (Fig. 1e).

At day 42, aggregates cultured in condition 2 had thin folded epithelium, a morphological characteristic seen in fetal choroid plexus epithelia (Fig. 1f). Immunohistochemistry (IHC) showed that transthyretin (TTR) signal was mainly seen apically in the thin NE portion (Fig. 1g,h; Supplementary Fig. 1d,e). In addition, other markers in choroid plexus epithelia were observed, such as Lmx1a, Otx2 and water channel Aquaporin-1 (Fig. 1g–j; Supplementary Fig. 1f–g), and tight junction marker ZO-1 was on the surface of epithelia (Fig. 1k). These characteristics in morphology and marker distribution were reminiscent of human fetal choroid plexus epithelia^15^.

To investigate whether other subtypes of BMP ligands combined with CHIR treatment could induce choroid plexus-like tissues, we used BMP 2 or BMP 7 treatment method instead of BMP4 in condition 2. Although a higher concentration was needed compared with BMP4, treatment with either BMP2 (200 ng ml^−l^) plus CHIR or BMP7 (600 ng ml^−1^) plus CHIR could induce choroid plexus-like tissues (Supplementary Fig. 1h–k, l–o).

Next, we examined the effect of applying CHIR and BMP4 independently. When CHIR were applied solely from day 18 to day 44 (condition 2 minus BMP4), hESC-derived aggregates had thick epithelium with attenuation of *Foxg1::*Venus expression (Supplementary Fig. 1p,q). The cortical hem markers Lmx1a and Otx2 were expressed in the epithelium (Supplementary Fig. 1r,s), though the choroid plexus marker TTR was not detected (Supplementary Fig. 1r), suggesting that hem-like tissue was mainly induced by CHIR-only treatment. In contrast, when hESC-derived cortical NE was treated with BMP4 alone from day 18 to day 44 (condition 2 minus CHIR), *Foxg1*::Venus expression was attenuated in the aggregates (Supplementary Fig. 1t,u) and the epithelium expressed Lmx1a and Otx2 (Supplementary Fig. 1v,w), with some TTR expression (Supplementary Fig. 1v), suggesting partial induction of choroid plexus.

Collectively, these results indicate that treatment with CHIR alone induced cortical hem-like tissues and treatment with BMP4 alone partially induced choroid plexus-like tissue, but treatment with both CHIR and BMP4 (condition 2) showed strong dorsalizing activity and dominantly induced choroid plexus-like tissues from hESCs in 3D culture.

### Induction of dorsomedial telencephalic tissues from hESCs

**Figure 2:**
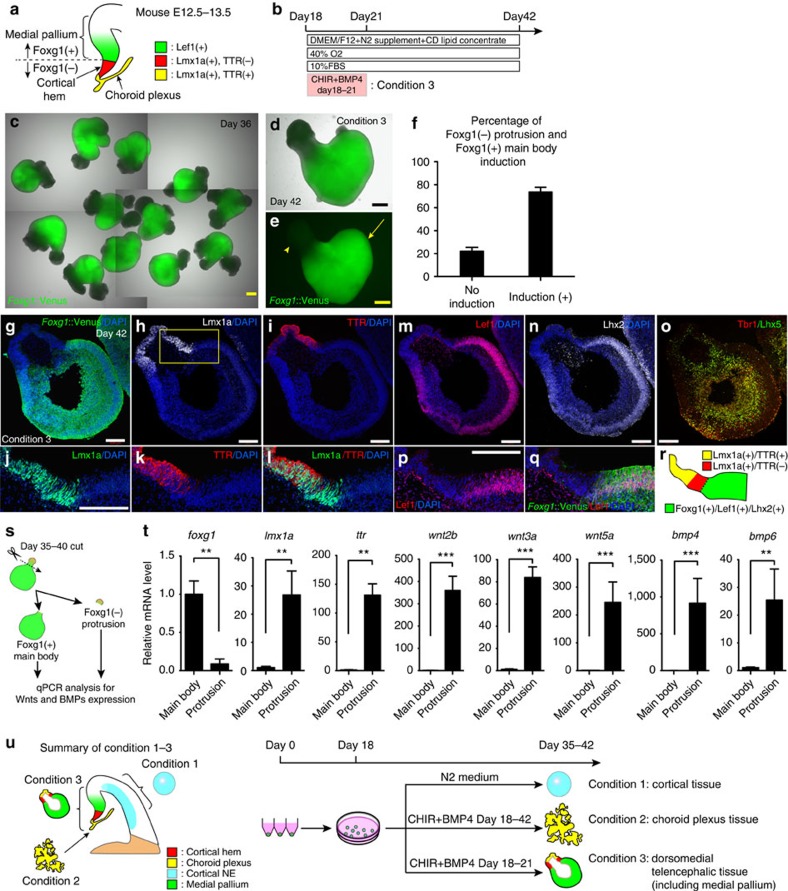
Transient exposure of dorsalizing factors can induce patterned dorsomedial telencephalic tissues. (**a**) Schematic of mouse medial pallium and neighbouring tissues at E12.5–13.5. (**b**) Schematic of condition to induce medial pallium tissues. (**c**) Bright-field view of aggregates cultured in condition 3 on day 36. (**d**,**e**) By shortening the period (day 18–21), the aggregates expressed *Foxg1*::Venus with patterning into *Foxg1::*Venus^+^ (arrow) and Venus^−^ (arrowhead) NE domains. (**f**) Histogram of percentage of *Foxg1*::Venus^−^ protrusion and *Foxg1*::Venus^+^ main body induction. Patterning of *Foxg1::*Venus^+^/Venus^−^ NE domains were induced in around 70–80% of aggregates (bars in graph, s.e.m.). (**g**–**l**) The Venus^−^ NE domains of aggregates (**g**) showed TTR^+^/Lmx1a^+^ in distal parts (**h**,**i**) and Lmx1a^+^/TTR^−^ in proximal parts (**j**–**l**, box in **h**). (**m**–**o**) In hESC-derived NE, *Foxg1*::Venus^+^ main bodies expressed Lef1 (**m**) and Lhx2 (**n**). (**o**) On the basal side, the thick cell layer of the aggregate expressed Tbr1 and Lhx5. (**p**) *Foxg1*::Venus and Lef1 co-expressed in continuous NE (**p**,**q**, box in **h**), suggesting medial pallium-like NE generation. (**r**) Schematic of hESC-derived dorsomedial telencephalic tissues. Choroid plexus-, hem- and medial pallium-like regions were continuously generated as seen *in vivo*. (**s**) Schematic of method to examine Wnts/BMPs expression in Venus^−^ protrusions. The aggregates were cut into Venus^−^ protrusions and Venus^+^ main bodies at day 35–40, and *wnts/bmps* expression were examined using RT–qPCR. (**t**) qPCR for genes expressed in Venus^−^ protrusion versus Venus^+^ main body (**P<0.01; ***P<0.001, *n*=3, unpaired *t*-test). *foxg1* was significantly attenuated in Venus^−^ protrusions, and a significant increase in *lmx1a/ttr* mRNA expression in Venus^−^ protrusions was confirmed. *wnt 2b/3a/5a* and *bmp 4/6* significantly increased in Venus^−^ protrusions. (**u**) Schematic summary of conditions examined in Figs 1 and 2. Scale bars, 500 μm (**c**–**e**); 200 μm (**g**–**p**). Bars in graph, s.e.m. Nuclear counter staining (blue), 4,6-diamidino-2-phenylindole (DAPI).

Since the medial pallium is the precursor to the hippocampus^3^, and the hem is known to function as a hippocampal organizer^16^, we next investigated whether hem and medial pallium tissues, which are located adjacent to choroid plexus (Fig. 2a), could be induced by modifying culture conditions. Since Foxg1 was expressed from medial pallium to neocortex, but not in choroid plexus and hem (Fig. 2a; Supplementary Fig. 1a)^17^, we tried to define the condition that produces aggregates with patterning into *Foxg1::*Venus^+^ (estimated as the medial pallium region) and Venus^−^ (estimated as the choroid plexus or hem region) NE domains. We adjusted the timing of the dorsalizing treatment, the concentration of CHIR or BMP4, and the treatment period. These experiments revealed that CHIR+BMP4 exposure during a specific time window was critical for induction of the aggregates with patterning into *Foxg1::*Venus^+^ and Venus^−^ NE domains (Fig. 2b–e; Supplementary Fig. 2a). As the exposure period was shortened, *Foxg1*::Venus expression increased (Supplementary Fig. 2d). We also noticed that the domain of Venus^−^ NE generally formed protrusions in the aggregates (Fig. 2d,e, arrowhead), and the main body of the aggregates expressed *Foxg1*::Venus (Fig. 2e, arrow). Since the 3-day treatment (days 18–21) was sufficient to induce the patterned expression of *Foxg1::*Venus in 70–80% of aggregates (Fig. 2b–f, condition 3), we focused on this condition for more in-depth analysis.

In mouse telencephalon, both choroid plexus and hem were marked by Lmx1a^18^, and the former was distinguished from the latter by TTR expression (Fig. 2a; Supplementary Fig. 2b). Msx1 and 2, both of which are downstream targets of BMPs, are expressed in the dorsal midline of the telencephalon^5^. With this in mind, we first examined the gene expression characteristics of the *Foxg1*::Venus^−^ portion. IHC showed that the distal ends of Venus^−^ NE protrusions (Fig. 2g) were Lmx1a^+^ (Fig. 2h) and TTR^+^ (Fig. 2i). Notably, the proximal part of the protrusions was Lmx1a^+^ but TTR^-^ (Fig. 2j–l), resembling the marker expression pattern of the embryonic cortical hem (Supplementary Fig. 2b)^11^. In addition, Msx1/2 was also detected in the Venus^−^ NE protrusions (Supplementary Fig. 2e,f). These observations demonstrate that a 3-day exposure to the dorsalizing factors permitted the formation of a hem-like region next to the choroid plexus in the *Foxg1*::Venus^−^ protrusions of hESC-derived NE.

Then, we sought to further define the character of the *Foxg1*::Venus^+^ domain. Because it is well known that hem and choroid plexus function as a dorsalizing organizer of telencephalon to pattern the medial pallium^16^,^19^, we wondered whether the remaining *Foxg1*::Venus^+^ domain might contain medial pallium. In mouse dorsal telencephalon, medial pallium was marked by Lef1 and Lhx2 expression (Fig. 2a; Supplementary Fig. 2c)^14^. Consistent with this, the Venus^+^ main bodies of aggregates expressed Lef1 and Lhx2 (Fig. 2m,n; Supplementary Fig. 2g), with some variations in structures (Supplementary Fig. 2g–h). In the aggregates cultured under condition 3, almost all of the NE in the Venus^+^ main body expressed Lef1/Lhx2/Pax6, and lateral ganglionic eminence marker Gsh2 expression was rarely seen (Supplementary Fig. 2i–j), suggesting that the Venus^+^ main body of these aggregates mainly consisted of medial pallium. When CHIR plus BMP4 treatment was removed from condition 3, aggregates had a medial pallium portion (Lef1^+^/Lhx2^+^/Pax6^+^), a dorsolateral pallium portion (Lef1^−^/Lhx2^+^/Pax6^+^) and an lateral ganglionic eminence portion (Lhx2^+^/Pax6^−^/Gsh2^+^) (Supplementary Fig. 2k–n), forming the D–V axis in one aggregate (Supplementary Fig. 2o). Thus, the aggregates that had the potential to become ventral-to-dorsal pallium were actually dorsalized by treatment with CHIR plus BMP4, resulting in the dominant induction of medial pallium-like tissues in the Venus^+^ main body.

The apical side (aPKC^+^) of the NE was located on the surface of the aggregate (Supplementary Fig. 2p), and to the basal side, a thick cell layer expressed Tbr1 (Fig. 2o; Supplementary Fig. 2q), suggesting Lef1^+^ NE produced postmitotic neurons. Moreover, this cell layer also expressed Lhx5 (Fig. 2o; Supplementary Fig. 2r), one of the markers for Cajal–Rezius cells^20^. The Lef1^+^/*Foxg1*::Venus^+^ NE (Fig. 2p,q) formed a continuous structure next to Lmx1a^+^/*Foxg1*::Venus^−^ NE (Supplementary Fig. 2s–u), suggesting the generation of patterned dorsomedial telencephalic tissues that contain choroid plexus-, hem- and medial pallium-like tissues, as seen *in vivo* (Fig. 2r; Supplementary Fig. 2v).

Next, we investigated whether a Venus^−^ protrusion, which corresponds to cortical hem and choroid plexus, has the ability to produce Wnts and BMPs, as seen *in vivo*. Towards this aim, we cut the aggregate into a *Foxg1*::Venus^−^ protrusion and a *Foxg1*::Venus^+^ main body at day 35–40, and checked for *wnt 2b/3a/5a* and *bmp 4/6* expression using RT–qPCR (Fig. 2s). We found a significant decrease in *foxg1* mRNA expression and a significant increase of *lmx1a/ttr* mRNA expression in Venus^−^ protrusions (Fig. 2t). Notably, *wnt 2b/3a/5a* and *bmp 4/6* mRNA expression were significantly higher in Venus^−^ protrusions (Fig. 2t). This result supports the possibility that Venus^−^ protrusions actually possess the ability to secrete dorsalizing factors that participate in patterning the dorsomedial telencephalon *in vivo*.

Taken together, these findings demonstrate that our culture strategy of ‘transient CHIR plus BMP4 treatment' of cortical tissues (condition 3) could recapitulate the *in vivo*-like regional pattern formation of the dorsomedial telencephalon, thus allowing the self-organizing emergence of medial pallium tissues from hESCs (Fig. 2u; Supplementary Fig. 2v).

### *In vitro* generation of hippocampal primordium-like tissue

In the developing embryo, hippocampus arises from medial pallium. Thus, we further cultured our hESC-derived medial pallium tissues to examine whether hippocampal primordial tissues would develop. Since the NE of aggregates tended to get disorganized into rosette-like structures within 50 days (Supplementary Fig. 3a), it was challenging to retain continuous NE for a longer period. However, we found that by changing the basal media to Neurobasal medium at day 27, half-cutting the aggregates at day 35, and transferring the aggregates onto dishes with high O_2_-penetrating bottoms (Lumox dish) from day 50, we could maintain a continuous NE structure until culture day 75 (Supplementary Fig. 3b).

**Figure 3:**
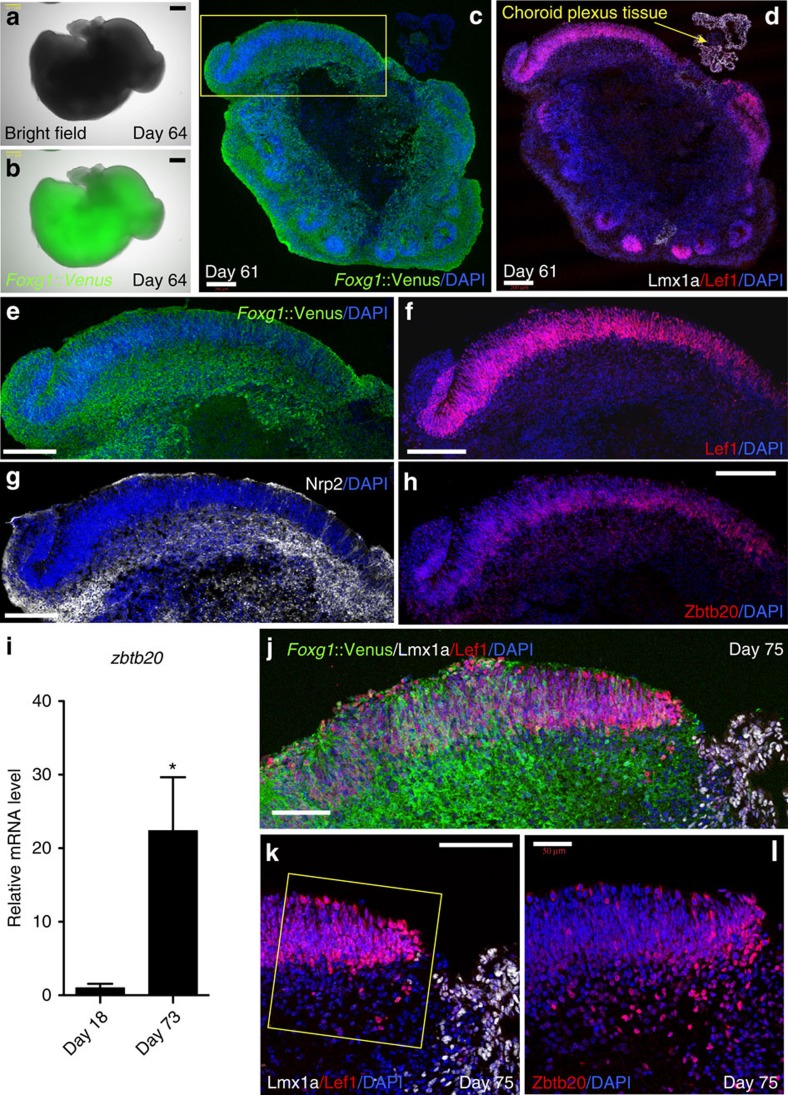
Expression of hippocampus marker Zbtb20 in medial pallium-like tissue. Under the optimized conditions, the aggregate was cultured with less formation of rosette-like NE (**a**,**b**). (**e**–**h** is box in **c**) Immunostaining on day 61 showed that the medial pallium-like continuous NE, positive for *Foxg1*::Venus (**e**) and Lef1 (**f**) were formed adjacent to the Lmx1a^+^ choroid plexus-like domain (**c**,**d**). (**g**) The more basal layer of the Lef1^+^ NE was positive for Nrp2. (**h**) Zbtb20 was expressed in the Lef1^+^ NE. (**i**) Zbtb20 mRNA expression at day 73 showed a significant increase compared with day 18. *P<0.05, *n*=3, Unpaired *t*-test with Welch's correction. (**j**) The medial pallium-like continuous NE, positive for *Foxg1*::Venus and Lef1 were clearly formed adjacent to the Lmx1a^+^ choroid plexus-like domain even at day 75. (**k**,**l**) Zbtb20 expression in cells beneath the Lef1^+^ NE was clearly seen at day 75 (**l** is enlarged box in **k**). Scale bars, 300 μm (**a**,**b**); 200 μm (**c**–**h**); 100 μm (**j**,**k**); 50 μm (**l**). Bar in graph, s.e.m. Nuclear counter staining (blue), 4,6-diamidino-2-phenylindole (DAPI).

Under these conditions, the aggregates had less rosette-like NE (Fig. 3a,b), and on day 61, Lef1^+^/*Foxg1*::Venus^+^ medial pallium-like continuous NE portions clearly formed adjacent to the Lmx1a^+^ choroid plexus- and hem-like domain (Fig. 3c–f). Importantly, a more basal layer of the Lef1^+^ NE was positive for Nrp2 (neuropillin 2) (Fig. 3g), as seen in the embryonic hippocampus^21^. In addition, the Lef1^+^ NE expressed Zbtb20 (BTB/POZ zinc-finger family; Fig. 3h), a marker for hippocampal neurons and their precursors (Supplementary Fig. 3c)^22^. This was also confirmed using RT–qPCR (Fig. 3i). Even in culture for over 70 days, Lef1^+^/*Foxg1*::Venus^+^ continuous NE clearly formed adjacent to the Lmx1a^+^ /*Foxg1*::Venus^−^ domain (Fig. 3j,k). IHC showed clear Zbtb20 expression in cells beneath the Lef1^+^ NE (Fig. 3l), and weak Zbtb20 expression in the NE (Fig. 3l). Collectively, these results demonstrate the successful *in vitro* generation of hippocampal primordium-like tissue.

### Generation of hippocampal pyramidal- and granule-like neurons

We next asked whether the hippocampal primordium-like tissue could generate the specific neuronal subtypes found in the hippocampus *in vivo*. Hippocampus is divided into distinct regions *in vivo*: CA1 and CA3 of Ammon's horn, and the dentate gyrus (DG) (Supplementary Fig. 4a)^2^. CA fields and DG consist of neurons with large pyramidal somata and small granule somata, respectively. Besides the cell morphology, it is known that DG granule neurons express Prox1 (prospero-related homeobox 1), while CA3 pyramidal neurons express KA1 (kainate acid 1, also known as grik4: glutamate receptor ionotropic kainate 4; Supplementary Fig. 4a–b)^2^,^19^.

**Figure 4:**
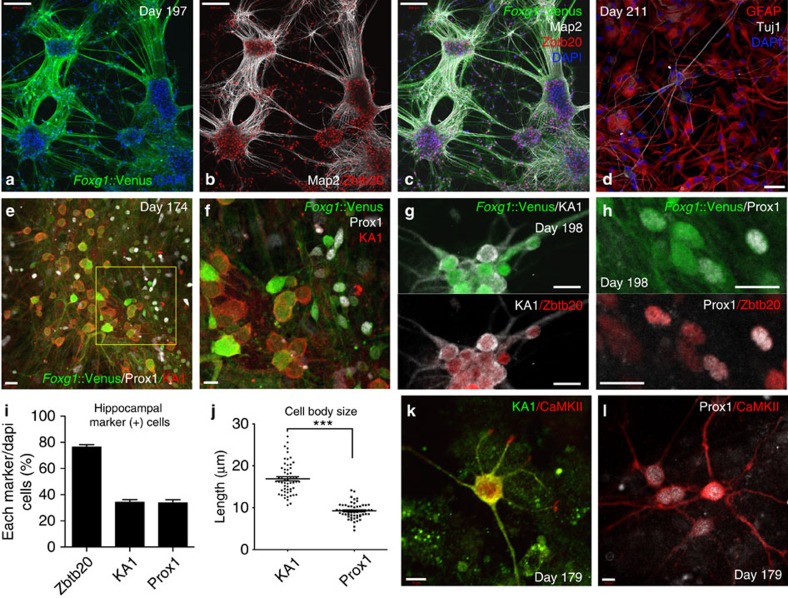
Hippocampal marker-positive pyramidal and granule-like neurons were generated by long-term dissociation culture. (**a**–**c**) Immunostaining of dissociated cells at day 197. *Foxg1*::Venus^+^ neurons (**a**) were connected to one another by MAP2^+^ neurites (**b,c**). GFAP^+^ glial-like cells were also detected (**d**). (**e**,**f**) KA1^+^/*Foxg1*::Venus^+^ neurons (CA type) tended to have large cell bodies; in contrast, Prox1^+^/*Foxg1*::Venus^+^ neurons (DG type) mostly had small somata (**f** is enlarged box in **e**). Zbtb20 was co-expressed with both KA1^+^ and Prox1^+^ neurons (**g**,**h**). (**i**) The percentage of Zbtb20^+^ cells was about 75% (means: 76.5%, s.e.m.: 1.65) and the percentages of KA1^+^ (means: 34.4%, s.e.m.: 1.83) and Prox1^+^ (means: 33.8%, s.e.m.: 2.33) were both about 34%. Countered neurons: Zbtb20^+^ (*n*=2764), KA1^+^ (*n*=147), Prox1^+^ (*n*=188). (**j**) Cell body size was significantly larger in KA1^+^ cells. ***P<0.001, paired *t*-test. Countered neurons: KA1^+^ (*n*=56), Prox1^+^ (*n*=55). (**k**,**l**) CaMKII was expressed in KA1^+^ (**k**) and Prox1^+^ (**l**) neurons. Scale bars, 200 μm (**a**–**c**); 50 μm (**d**); 20 μm (**e**,**g**–**h**); 10 μm (**f**,**k**,**l**). Bars in graph, s.e.m. Nuclear counter staining (blue), 4,6-diamidino-2-phenylindole (DAPI).

We first tried further long-term culture to assess CA and DG fields, but the organoids could not be maintained beyond day 100 in a healthy condition. Thus, to evaluate the neuronal population and function of the hESC-derived dorsomedial telencephalic tissues, we utilized a dissociation culture that enables long-term culture and facilitates neuronal maturation. Cells were dissociated at days 73–84 and plated on poly-D-lysine-laminin-coated plates (Supplementary Fig. 4c). At day 197 (17 weeks after dispersion), these cells made multiple small aggregations (Fig. 4a), which were connected to one another by numerous MAP2^+^ neurites (Fig. 4b). The majority of the cells expressed Zbtb20 (Fig. 4b,c). Unexpectedly, we also detected *Foxg1*::Venus^+^/GFAP^+^ glial-like cells (Fig. 4d; Supplementary Fig. 4d–g). Other markers for astrocytes such as S100 beta^23^ and vimentin^24^ were also detected in the glial-like cells (Supplementray Fig. 4h–k,l–o). These data demonstrate the generation of hippocampal-like neurons and astrocyte-like cells from hESC-derived dorsomedial telencephalic tissues.

We next examined the hippocampal regional marker expression using immunocytochemistry. These analyses showed the existence of KA1^+^/*Foxg1*::Venus^+^ neurons and Prox1^+^/*Foxg1*::Venus^+^ neurons (Fig. 4e,f). KA1^+^ and Prox1^+^ neurons co-expressed Zbtb20 (Fig. 4g,h), suggesting these neurons had CA- and DG-type features, respectively. The percentage of Zbtb20^+^ cells was ∼75%, and KA1^+^ and Prox1^+^ cell percentages were each ∼34% (Fig. 4i). Cell body size was significantly larger in KA1^+^ CA-type neurons (Fig. 4j, *P*<0.001, paired *t*-test), consistent with previous *in vivo* analysis of human hippocampal neurons^25^. Moreover, CaMKII, one of the maturation markers of hippocampal neurons^26^, was expressed in most KA1^+^ and Prox1^+^ neurons (Fig. 4k,l). These observations suggest the emergence of hippocampal pyramidal- and granule-like neurons from hESC-derived dorsomedial telencephalic tissues, which led us to further functional examinations.

**Figure 5:**
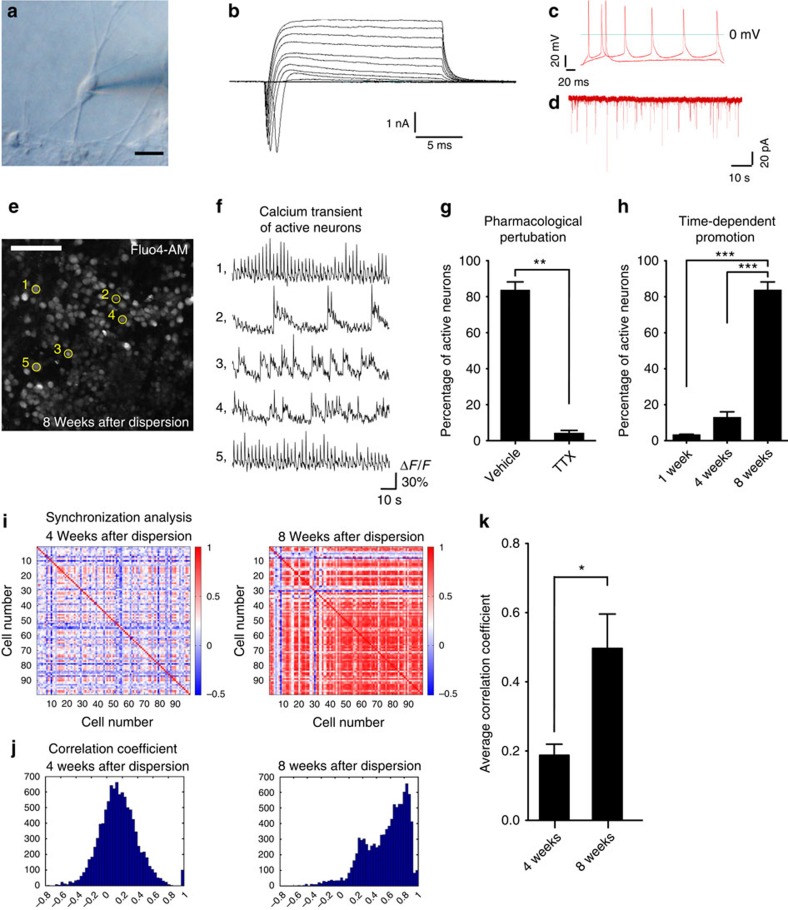
Functional analysis of dissociated hippocampal-like neurons using electrophysiology and calcium imaging. (**a**) A representative cell image for patch-clamp recording. Many neurons showed voltage-dependent Na–K current (**b**, *n*=30), action potential following injection of depolarizing currents (**c**, *n*=18) and spontaneous excitatory postsynaptic currents (**d,** *n*=9, 2.33±0.75 Hz), at day 136 (dissociated at day 83). The percentage of neurons recorded showing Na–K currents, action potentials and synaptic responses were 100% (30 out of 30), 72% (18 out of 25) and 75% (9 out of 12), respectively. (**e**–**h**) The data set of calcium imaging. (**e**) Representative image of active neurons (day 143, 8 weeks after dissociation), and their firing pattern shown by trace image of calcium response (**f**). (**g**) Pharmacological perturbation by TTX (**P<0.01, *n*=3, unpaired *t*-test with Welch's correction). (**h**) Time-dependent promotion of neuronal activity. Percentage of active neurons significantly increased at 8 weeks after dissociation (***P<0.001, *n*=3, one-way analysis of variance). (**i**) Synchronization analysis by cross-correlations of 100 neurons at 8 and 4 weeks after dissociation. Colours from red to blue indicate their cross-correlations from high (synchronized activity) to low. Cross-correlation was higher at 8 weeks after dissociation than 4 weeks. The data are representative of three independent experiments. (**j**) Histogram of correlation coefficients indicated a strong correlation at 8 weeks after dissociation. The data are representative of three independent experiments. (**k**) Histogram of average correlation coefficients of one hundred neurons (*P<0.05, *n*=3, unpaired *t*-test). Scale bars, 20 μm (**a**); 100 μm (**e**). Bars in graph, s.e.m.

For the analysis of synaptic formation, we first performed whole-cell patch-clamp recording of hippocampal pyramidal-like neurons at day 136 (7 weeks after dispersion) (Fig. 5a). The resting membrane potentials were −58.7±3.0 mV (*n*=13). We observed that the cultured neurons had voltage-dependent Na–K current (Fig. 5b), action potential following injection of depolarizing currents (Fig. 5c), and spontaneous excitatory postsynaptic currents (Fig. 5d, 2.33±0.75 Hz), which were blocked by bath application of CNQX, an antagonist of non-*N*-methyl-D-aspartate receptors (Supplementary Fig. 5a). These results suggest that the neurons were mature enough to form functional synapses.

Next, to evaluate the functional network formation of the neurons, we examined intracellular calcium dynamics by live imaging. On day 143 (8 weeks after dissociation), individual neurons showed spontaneous calcium transients (Fig. 5e,f). These transients were inhibited by the Na^+^ channel blocker tetrodotoxin (TTX; Fig. 5g) and recovered after ceasing TTX treatment (Supplementary Fig. 5b–c; Supplementary Movie 1), attesting to their correlation with neuronal activity. The percentage of active neurons significantly increased at 8 weeks after dissociation versus 1 or 4 weeks after dissociation of ES cell-derived hippocampal precursors, suggesting a time-dependent promotion in the spontaneous neuronal activity (Fig. 5h). Notably, calcium transients at 8 weeks after dissociation showed a synchronized pattern of network activity that was not seen at 4 weeks after dissociation (Supplementary Fig. 5d–e; Supplementary Movie 2). Cross-correlation analysis showed that the rate of synchronized calcium transients was significantly higher at 8 weeks after dissociation than 4 weeks (Fig. 5i–k). These data serve as further evidence for the development of synaptic connections and a neuronal network^27^,^28^.

Taken together, our functional analysis data showed that hippocampal granule- and pyramidal-like neurons generated from hESC-derived medial pallium-like tissues had functional neuronal activity with synchronization *in vitro*.

## Discussion

In this report, we have demonstrated the 3D generation of choroid plexus- and medial pallium-like tissues from hESCs through a modified neocortical induction method (Fig. 2u)^11^,^12^. After long-term culture, hESC-derived medial pallium tissues gave rise to Zbtb20^+^ hippocampal neurons. Prox1^+^ granule- and KA1^+^ pyramidal-like neurons were contained within this population, suggesting the generation of DG- and CA-type neurons. These neurons formed a neuronal network with synaptic connections, as evidenced by their functional activity and CaMKII expression.

The critical piece of our method is treatment with two exogenous dorsalizing factors, CHIR and BMP4. With these, we started to explore the conditions suitable for choroid plexus generation. Our differentiation method (condition 2 in Fig. 1) led to the emergence of papillary thin TTR^+^ choroid plexus tissues in 3D-floating culture with high efficiency, showing some advantages over the previous induction protocol of choroid plexus tissue from hESC^29^. Choroid plexus and its derivative cerebrospinal fluid are thought to play important roles in nervous system function and several diseases such as Alzheimer's disease^30^. Since the investigation of human tissue is not easy, our culture protocol may provide a new tool for human choroid plexus research *in vitro*.

Our culture system was also used to induce medial pallium tissues by optimizing the CHIR and BMP exposure period to 3 days (days 18–21; condition 3 in Fig. 2). Under this condition, the aggregates expressed *Foxg1*::Venus with characteristic patterning; that is, *Foxg1::*Venus^+^/ Lhx2^+^ /Lef1^+^ domains were juxtaposed with Venus^−^ NE domains. Importantly, the marker expression separated the Venus^−^ domains into two regions that were reminiscent of choroid plexus and hem, respectively. From these results, we demonstrated the formation of continuous epithelium that consisted of choroid plexus-, hem- and medial pallium-like tissues as seen *in vivo*.

The underlying mechanism that induces this continuous dorsomedial structure is intriguing. We speculated a possible mechanism: (i) a small portion of hESC-derived cortical NE differentiated into *Foxg1*::Venus^−^ dorsomedial tissues by culturing in the medium for choroid plexus induction (days 18–21); (ii) despite culturing in CHIR/BMP4-free medium (based on the conditions for neocortex induction) from day 21, a Venus^−^ domain committed to dorsomedial tissues remained and functioned as a dorsalizing organizer (Fig. 2s,t); (iii) with the effect of a Venus^−^ domain, *Foxg1*::Venus^+^ domain expressed Lef1 and Lhx2, which resulted in dorsomedial pattern formation as seen *in vivo* (day 21–42). Previous *in vivo* studies have revealed the sufficiency of dorsomedial tissues for patterning the dorsal telencephalon^31^, and to date, several genes, such as Lhx2 (refs 16, 32), Lhx5 (ref. 33), Lef1 (ref. 34), Prox1 (ref. 35) and Zbtb20 (ref. 22), have been reported to affect hippocampal developmental patterning and/or proliferation. How these genes contribute to pattern formation in our system is an interesting avenue for future investigation.

In mouse hippocampus, Zbtb20 (whole hippocampal marker) and Prox1 (DG marker) start to express from E12.5, and then KA1 (CA3 marker) is expressed from E14.5 (Supplementary Fig. 4p). CaMKII expression slowly increases during postnatal development. In our method, Zbtb20 was confirmed as early as day 60 s, and Prox1 and KA1 were detectable from day 70 s and day 100 s, respectively. CaMKII was first detected at day 170 s. As a developmental model, this sequential order of hippocampal marker expression suggests that our method recapitulates embryonic developmental stages of hippocampus and generation of hippocampal neurons (Supplementary Fig. 4p). Unfortunately, however, due in part to the limitation of long-term 3D culture to form the whole hippocampal region, we were unable to generate CA1 pyramydal neurons and to recapitulate DG formation or regionally specified hippocampal tissues. A future investigation of hPSC-derived hippocampal induction would also focus on these issues.

There have been many reports regarding spontaneous development of synchronous activity during cortical network formation *in vitro*^36^. It occurs during early central nervous system development, and is thought to play an important role in neuronal development, formation of neuronal circuits, neuronal information processing and phenomena occurring during epileptic seizures^37^,^38^. To our knowledge, this is the first report of synchronous calcium transients in hPSC-derived neuronal tissues. One factor that enables this synchronous calcium transient in our system might be the simultaneous generation of astrocytes, which is thought to be essential for neuronal oscillatory activity^39^. Synchronous neuronal firing is a transient phenomenon, and later as the developmental stages proceed, more complex firing patterns replace it^40^. Therefore, our culture system may represent an early hippocampal neuronal developmental stage. The exploration of firing pattern formation in hPSC-derived neuronal tissues is a future topic that might be useful for research relating to human brain function or functional neuronal diseases such as epileptic seizure.

The hippocampus has also been implicated in several neuropsychiatric disorders, including Alzheimer's disease and schizophrenia^41^. Exploring any of the molecular mechanisms related to these disorders, however, has traditionally been a challenge. For this reason, induction of hippocampal neurons from hPSCs should provide a new means to research the molecular biology of hippocampus-relevant disorders. Towards this aim, Yu *et al.* first reported a protocol for selective differentiation of DG granule neurons from hPSCs^42^. They differentiated DG granule neurons using Wnt3a/BDNF treatment and maintaining them on co-culture with hippocampal astrocytes. However, the generation of a hippocampal region in 3D order, glial cells and CA pyramidal neurons has not yet been attained. In contrast, our system provides dorsomedial telencephalon-like 3D tissues that give rise to hippocampal granule- (DG) and pyramidal- (CA) like neurons, as well as astrocyte-like cells. Since hippocampal neurons form neural circuitry between different types of neurons (tri-synaptic circuits for instance), recapitulation of this complex neural circuitry will be necessary for future medical research relevant to the hippocampus^43^. Thus, our approach provides a new experimental platform for the analysis of human hippocampus-related disorders.

## Methods

### Maintenance culture of hESCs

Human ES cells (hESCs; KhES-1, RIKEN BioResource Center, Cell Number: HES0001) were used according to the hESC research guidelines of the Japanese government. hESCs were maintained and cultured as previously described^12^. In brief, hESCs were maintained on a feeder layer of mouse embryonic fibroblasts inactivated by mitomycin C treatment in DMEM/F-12 (Sigma) supplemented with 20% (vol/vol) Knockout Serum Replacement (KSR; Invitrogen), 2 mM glutamine, 0.1 mM nonessential amino acids (Invitrogen), 5 ng ml^−1^ recombinant human basic fibroblast growth factor (FGF) (Wako), 0.1 mM 2-mercaptoethanol, 50 U ml^−1^ penicillin and 50 μg ml^−1^ streptomycin under 2% CO_2_. For passaging, hESC colonies were detached and recovered en bloc from the feeder layer by treating them with 0.25% (wt/vol) trypsin and 1 mg ml^−1^ collagenase IV in PBS containing 20% (vol/vol) KSR and 1 mM CaCl_2_ at 37 °C for 7–8 min. The detached hESC clumps were broken into smaller pieces (several dozens of cells) by gentle pipetting. The passages were performed at a 1:4**–**1:6 split ratio every 4–5 days.

### Differentiation culture of hESCs

For SFEBq culture, hESCs were dissociated to single cells in TrypLE Express (Invitrogen) containing 0.05 mg ml^−1^ DNase I (Roche) and 10 μM Y-27632 (TOCRIS), and quickly reaggregated using low-cell-adhesion 96-well plates with V-bottomed conical wells (Sumilon PrimeSurface plate; Sumitomo Bakelite) in differentiation medium (9,000 cells per well, 100 μl) containing 20 μM Y-27632 under 5% CO_2_. The differentiation medium was Glasgow-MEM (Invitrogen) supplemented with 20% (vol/vol) KSR, 0.1 mM nonessential amino acids, 1 mM pyruvate, 0.1 mM 2-mercaptoethanol, 100 U ml^−1^ penicillin and 100 μg ml^−1^ streptomycin. Defining the day on which the SFEBq culture was started as day 0, 3 μM IWR1e (Wnt inhibitor; Calbiochem) and 5 μM SB431542 (TGFβ inhibitor; TOCRIS) were added to culture from day 0 to day 18. The culture method after day 18 was as follows.

For neocortical induction (condition 1, Fig. 1)^12^, at day 18, the floating aggregates were transferred from a 96-well plate to a 9-cm Petri dish (non-cell adhesive) and further cultured in suspension using DMEM/F-12, GlutaMAX(TM) (Gibco) medium supplemented with 1% N-2 supplement (Invitrogen), 1% Chemically Defined Lipid Concentrate (Invitrogen), 0.25 mg ml^−1^ fungizone (Gibco), 100 U ml^−1^ penicillin and 100 μg ml^−1^ streptomycin under 40% O_2_/5% CO_2_ conditions.

For choroid plexus-like tissue generation (condition 2, Fig. 1), from day 18, the floating aggregates were transferred to a Petri dish (non**-**cell adhesive) and further cultured in suspension using DMEM/F-12 GlutaMAX(TM) (Gibco) supplemented with 1% N-2 supplement (Invitrogen), 1% Chemically Defined Lipid Concentrate (Invitrogen), fetal bovine serum (FBS; 10% vol/vol), 3 μM CHIR 99021 (GSK3 inhibitor; Stemgent) and 0.5 nM BMP4 (R&D) under 40% O_2_/5% CO_2_ conditions. Medium was changed once every 3 days.

For medial pallium induction (condition 3, Fig. 2), the culture condition from day 0 to day 21 was the same as the choroid plexus induction condition. On day 21, the medium was changed to DMEM/F-12 GlutaMAX(TM) supplemented with 1% N-2 supplement, 1% Chemically Defined Lipid Concentrate, and FBS (10% vol/vol). Medium was changed once every 3 days.

For long-term culture of medial pallium tissues, the aggregates were cultured under the following conditions. On day 27, the base medium was changed to Neurobasal medium (Gibco) supplemented with 2% B-27 supplement without vitamine A (Gibco), 2 mM L-glutamine, 100 U ml^−1^ penicillin, 100 μg ml^−1^ streptomycin, 0.25 mg ml^−1^ fungizone (Gibco) and FBS (10% vol/vol). At day 35, the aggregates were cut into half-size with fine forceps under a dissecting microscope for the prevention of cell death in the central portions, and were cultured using a Lumox dish (SARSTEDT; high-O_2_ penetration) after day 50. Medium change was performed once every 3 days.

### Neuronal dissociation culture method

For the dissociation culture, cells were dissociated from aggregates using a Neural Tissue Dissociation Kit (Sumitomo Bakelite, MB-X9901) on days 73–84, and plated onto poly-D-lysine/laminin/fibronectin-coated glass dishes at a density of 300,000–500,000 cells per cm^2^ in Neurobasal medium supplemented with 2% B-27 supplement without vitamine A, 100 U ml^−i^ penicillin, 100 μg ml^−1^ streptomycin, 0.25 mg ml^−1^ fungizone and 10% FBS. The medium was changed every 3 days.

For electrophysiology and calcium imaging, the dissociation culture was modified slightly for suitability of examinations. Cells were dissociated on days 73–103 as described above, and plated onto poly-D-lysine/laminin/fibronectin-coated Cell Desk LF-1 (Sumitomo Bakelite, MS-92132) at a density of 300,000 cells per cm^2^ in Neurobasal medium (supplemented with 2% B-27 supplement without vitamine A, 20 ng ml^−1^ BDNF, 20 ng ml^−1^ NT-3, 5% astrocyte conditioned medium (Sumitomo Bakelite), 100 U ml^−1^ penicillin, 100 μg ml^−1^ streptomycin, 0.25 mg ml^−1^ fungizone, 1% FBS and 10 μM Y-27632). Three days after dissociation, half the medium was changed to Neurobasal supplemented with B-27 supplement without vitamine A, 20 ng ml^−1^ BDNF, 20 ng ml^−1^ NT-3, 5% astrocyte conditioned medium, 100 U ml^−1^ penicillin, 100 μg ml^−1^ streptomycin and 0.25 mg ml^−1^ fungizone. Afterwards, one-half of the medium volume was changed every 3 days.

### Immunohistochemistry and qPCR

IHC was performed as previously reported^12^. In brief, aggregates were fixed in 4% paraformaldehyde and incubated with primary antibodies (4 °C, overnight) following incubation with secondary antibodies conjugated with Alexa 488, 546 and 647 (room temperature, 2 h). Primary antibodies are described below. The antibodies were used at the following dilutions: GFP (rat, monoclonal, 1:500, Nakalai Tesque), Emx1 (gunia pig, 1:500, TaKaRa), Pax6 (rabbit, 1:250, Covance), TTR (rabbit, 1:1,000, DAKO), Aqp1 (rabbit, 1:500, Millipore), ZO-1 (rabbit, 1:100, Invitrogen), Lef1 (rabbit, 1:500, cell signalling), Lhx2 (goat, 1:100, Santa Cruz), Zbtb20 (rabbit, 1:200, Sigma-Aldrich), Prox1 (mouse, 1:200, Millipore) or (rabbit, 1:2000, Millipore), KA1(C-20) (goat, 1:100, Santa Cruz), MAP2 (mouse, 1:200, Sigma), GFAP (mouse, 1:200, Sigma), CaMKII (mouse, 1:500, Abcam) or (rabbit, 1:100, Santa Cruz), Otx2 (rabbit, 1:1,000, Abcam), Gsh2 (rabbit, 1:10,000, TaKaRa), (guinea pigs, 1:500, TaKaRa), Tuj1 (rabbit, 1:500, Covance), S100 beta (mouse, 1:500, Abcam), vimentin (mouse, 1:300, Abcam) and Msx1/2 (mouse, 1:200, DSHB). The antiserum specific to Lmx1a was raised in guinea pigs against a synthetic peptide (CFLATSEAGPLQSRVGNPIDHLYSMQNSYFTS; corresponding to the C-terminal residues 351**–**382 of the human Lmx1a protein) and was affinity purified (1:10,000–20,000). Counter nuclear staining was performed with 4,6-diamidino-2-phenylindole (Molecular Probes).

qPCR (6–8 aggregates per sample) was performed as previously described^10^, using a 7900 HT Fast Real Time PCR System (Applied Biosystems) and the data were normalized to the GAPDH expression. Primers were as follows:

*GAPDH*, forward 5′-TCAAGAAGGTGGTGAAGCAG-3′ reverse 5′CGCTGTTGAAGTCAGAGGAG-3′; *Foxg1*, forward 5′-GCCAGCAGCACTTTGAGTTA-3′, reverse 5′-GGTGGAGAAGGAGTGGTTGT-3′; *Lmx1a*, forward 5′-TCAGAAGGGTGATGAGTTTGTCC-3′, reverse 5′-GGGGCGCTTATGGTCCTTG-3′; *Otx2*, forward 5′-AGAGGACGACGTTCACTCG-3′, reverse 5′-TCGGGCAAGTTGATTTTCAGT-3′; *TTR*, forward 5′-ATCCAAGTGTCCTCTGATGGT-3′, reverse 5′-GCCAAGTGCCTTCCAGTAAGA-3′; *Zbtb20*, forward 5′-GAAACAGGTGCTTCCTCTCC-3′, reverse 5′-TTGACCGAAGGCTGTTGTAG-3′; *Wnt2b*, forward 5′-AGACACGTCCTGGTGGTACA-3′, reverse 5′-AACGCATGATGTCTGGGTAA-3′; *Wnt3a*, forward 5′-GATGGTGGTGGAGAAGCAC-3′, reverse 5′-GTGGGCACCTTGAAGTAGGT-3′; *Wnt5a*, forward 5′-CAACTGGCAGGACTTTCTCA-3′, reverse 5′-TTCTTTGATGCCTGTCTTCG-3′; *BMP4*, forward 5′-CTGGAATGACTGGATTGTGG-3′, reverse 5′-CATGGTTGGTTGAGTTGAGG-3′; *BMP6*, forward 5′-ACAGGAGCATCAGCACAGAG-3′; and reverse 5′-TAGTGGCCGTGATGTCAAAT-3′.

### Calcium imaging

For calcium dye loading, the cells were incubated with 5 μM Fluo4-AM solution (Invitrogen) for 40 min at 37 °C. Excess dye was removed by washing twice with culture medium. Imaging was carried out at 37 °C using an incubator-docked inverted microscope (LCV110, Olympus) equipped with a spinning disk confocal unit (CSU-W1, Yokogawa) and a high-sensitivity EM-CCD camera (iXon3, Andor) controlled by Metamorph software (Molecular Devices). Fluo4-AM dyes were excited at 488 nm using a diode-pumped solid-state laser (Sapphire, Coherent) and fluorescence emission was viewed through a silicon emersion objective (UPLSAPO30XS, Olympus). Time-lapse image sequences were acquired with 170-ms duration for 1 min. Images were processed using custom-designed Matlab (MathWorks) programs and Image J software. The fluorescence change over time is defined as Δ*F*/*F*=(*F*−*F*_basal_)/*F*_basal_, where *F* is the fluorescence at any time point and *F*_basal_ is the baseline fluorescence averaged across the whole movie for each cell. A neuron was considered as active if calcium transients were observed at least once per minute. For pharmacological experiments, TTX (1 μM, Alomone Labs) was applied by bath application or locally applied (3 μM) with Alexa 647-conjugated 10000MW dextran (Life Technologies) by pressure ejection (15–20 p.s.i. for 100 ms with 1-s interval) from glass pipettes positioned 250–500 μm from the cell of interest.

### Electrophysiology

Whole-cell patch-clamp recordings were performed for cells plated on poly-D-lysin/laminin/fibronetctin-coated Cell Desk LF-1 (Sumitomo Bakelite). All experiments were performed at room temperature. The chamber was constantly perfused at a rate of 2 ml min^−1^ with external solution containing (in mM) 140 NaCl, 2.5 CaCl_2_, 2 MgCl_2_, 10 glucose, 1 NaH_2_PO_4_ and 10 HEPES, pH 7.4 adjusted with NaOH. The micropipettes were made from borosilicate glass pipettes (Sutter Instrument, Novato, CA, USA, BF150-86-10) with micropipette puller (Sutter Instrument, P-1000). These pipettes had a resistance of 3–6 MΩ when filled with internal solution containing (in mM) 120 K gluconate, 10 KCl, 10 EGTA and 10 HEPES, pH 7.2 adjusted with KOH. Measurements were made using EPC-10 amplifiers (HEKA, EleKtronik, Lambrecht, Germany), low-pass filtered at 2.9 kHz, and digitized at 10 kHz for computer analysis with the PatchMaster software (HEKA) and LabChart software (AD Instruments, Bella VIsta, NSW, Australia). The series resistances were always <20 MΩ. The whole-cell capacitance was fully compensated. For voltage-clamp measurements, the membrane potential was held at −60 mV. Voltage pulses were injected into cells from −80 to 60 mV in 10-mV steps to monitor the sodium and potassium currents. For current-clamp measurement, cells were current clamped at around −60 mV, and monitored for current-induced action potentials. For pharmacological experiments, CNQX (10 μM, Sigma) was applied by bath application.

### Statistical analysis

Statistical tests were performed with PRISM software (GraphPad, ver 5). Statistical significance was tested with the paired *t*-test (parametric) or unpaired *t*-test (nonparametric) or unpaired *t*-test with Welch's correction (nonparametric) for two-group comparison or with the one-way analysis of variance test (parameteric; versus the control group) for multiple-group comparison.

## Additional information

**How to cite this article:** Sakaguchi, H. *et al.* Generation of functional hippocampal neurons from self-organizing human embryonic stem cell-derived dorsomedial telencephalic tissue. *Nat. Commun.* 6:8896 doi: 10.1038/ncomms9896 (2015).

## Supplementary Material

Supplementary Information Supplementary Figures 1-5

Supplementary Movie 1 Spontaneous calcium transients were inhibited by TTX local application and recovered after ceasing TTX treatment. Examination for intracellular calcium dynamics using Fluo4-AM showed that individual neurons had spontaneous calcium transients that were inhibited by TTX local application and recovered after ceasing TTX treatment. Image shows pre (left), TTX local application (middle), and 10 minutes after ceasing TTX application (right).

Supplementary Movie 2 Calcium imaging of hESC-derived hippocampal neurons at 4 weeks and 8 weeks after dissociation. At 4 weeks after dissociation, individual neurons showed spontaneous calcium transients that differed from neuron to neuron in both frequency and duration, whereas calcium transients at 8 weeks after dispersion showed synchronized pattern of network activity.
