# Supplementary material for: Generation of functional hippocampal neurons from self-organizing human embryonic stem cell-derived dorsomedial telencephalic tissue
Source: Nat Commun. 2015 Nov 17;6:8896. doi: 10.1038/ncomms9896 (PMC4660208; doi:10.1038/ncomms9896)
Supplement: Supplementary Information — Supplementary Figures 1-5 [file ncomms9896-s1.pdf]

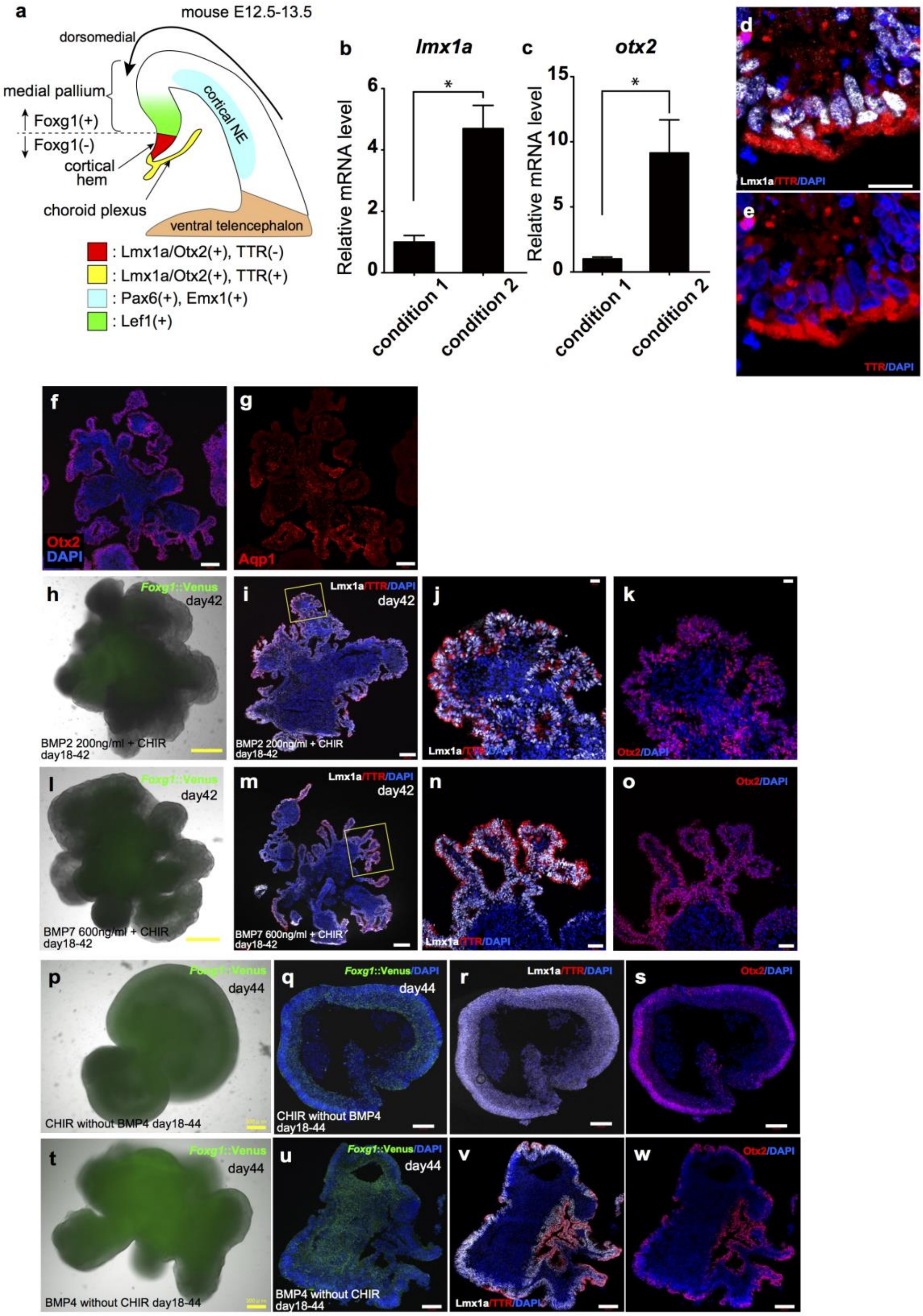

Supplementary Figure 1 | Generation of choroid plexus-like tissues with pleated structure from hESCs.

(Supplement to Fig. 1)

(**a**) Schematic of mouse dorsal telencephalon at E12.5-13.5. (**b-c**) qPCR for genes expressed in dorsomedial telencephalon. *$p<0.05$. In **b-c**, n=3; unpaired t-test with Welch's correction. *lmx1a* (**b**) and *otx2* (**c**) significantly upregulated in condition 2. (**d-g**) The induction of choroid plexus tissues with pleated structure from hESCs. Transthyretin (TTR) mainly stained apically (**d-e**). (**f-g**) The pleated epithelia are Otx2^{+} (**f**), and Aquaporin-1^{+} (**g**). (**h-o**) The investigation of other subtypes of BMP ligands combined with CHIR to induce choroid plexus-like tissues. Treatment with either BMP2 (200ng/ml) plus CHIR (**h-k**) or BMP7 (600ng/ml) plus CHIR (3μM) (**l-o**) could induce choroid plexus-like tissues as by BMP4 (0.5nM) plus CHIR (3μM) treatment. (**p-w**) The examination for the effect of applying CHIR and BMP4 independently. When CHIR (3μM) were applied solely from day 18 to day 44 (condition 2 minus BMP4), hESC-derived aggregate had thick epithelium with attenuation of *Foxg1*::Venus expression (**p,q**). Lmx1a and Otx2 were expressed in the epithelium (**r,s**), though TTR was not detected (**r**), suggesting hem-like tissue was mainly induced by CHIR-only treatment. When hESC-derived cortical neuroepithelium (NE) were treated with BMP4 (0.5nM) alone from day 18 to day 44 (condition 2 minus CHIR), *Foxg1*::Venus expression was attenuated in the aggregates (**t,u**) and the epithelium expressed Lmx1a and Otx2 (**v,w**), with TTR expression in some part (**v**), suggesting partial induction of choroid plexus. Scale bars, 500 μm in (**h, l**), 300 μm in (**p, t**), 200 μm in (**f-g, i, m, u-w**), 50 μm in (**n-o**), and 20 μm in (**d, j-k**). Bars in graph, SEM. Nuclear counter-staining (blue), DAPI.

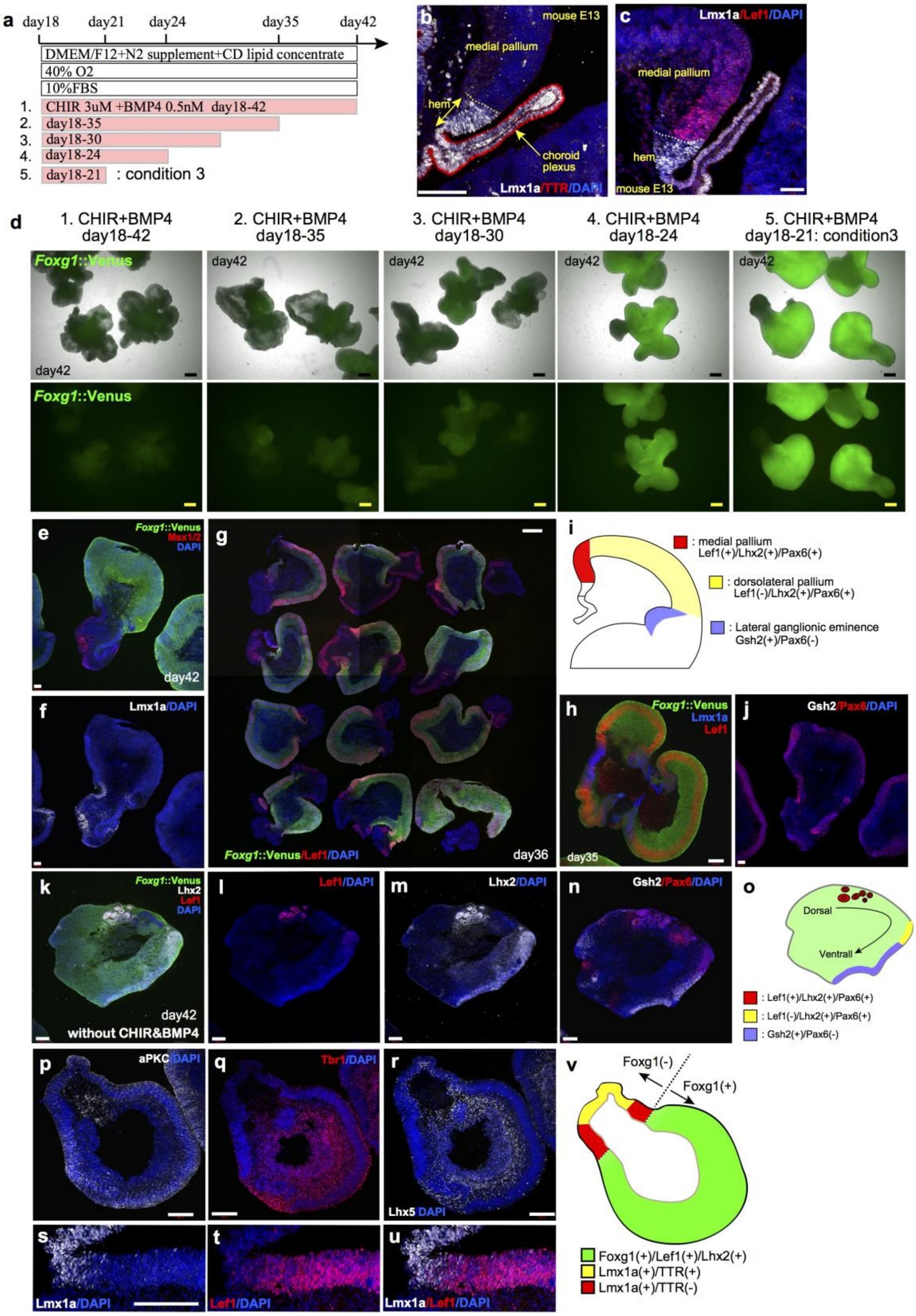

Supplementary Figure 2 | Transient exposure of dorsalizing factors can induce continuous structure containing choroid plexus-, hem-, and medial pallium-like tissues.

(Supplement to Fig. 2)

(**a**) Schematic of examined conditions to induce medial pallium tissues. (**b**) Immunostaining of mouse hem and choroid plexus at E13. (**c**) Immunostaining of mouse medial pallium at E13. (**d**) By shortening the period, the aggregates tended to express *Foxg1*::Venus with patterning into Venus^{+} and Venus^{-} NE domains (day 18-21 or day 18-24). IHC showed that the distal ends of Venus^{-} NE protrusions were Msx1/2^{+} and Lmx1a^{+} (**e-f**). The Venus^{+} main bodies of aggregates expressed *Foxg1*::Venus and Lef1 (**g**). These aggregates had variation in structure; in some aggregates, *Foxg1*::Venus^{+} NE grew large and the Lmx1a^{+} regions (hem plus choroid plexus) were found like the bottoms of furrows and flanked by curved NE that were *Foxg1*::Venus^{+}/Lef1^{+} (**h**). (**i**) Schematic of marker expression in mouse telencephalon including medial pallium, dorsolateral pallium, and lateral ganglionic eminence (LGE). In the aggregates cultured under condition 3, almost all of NE in Venus^{+} main body expressed Lef1/Pax6, and Gsh2 expression was rarely seen (**e-j**). When CHIR plus BMP4 treatment was removed from condition 3, aggregates had a medial pallium portion (Lef1^{+}/Lhx2^{+}/Pax6^{+}), a dorsolateral pallium portion (Lef1^{-}/Lhx2^{+}/Pax6^{+}), and an LGE portion (Lhx2^{+}/Pax6^{-}/Gsh2^{+}) (**k-n**), forming the D-V axis in one aggregate (**o**). (**p**) The aPKC^{+} apical side located surface of the aggregate. (**q-r**) To the basal side, the thick cell layer of the aggregate expressed Tbr1 and Lhx5. Lef1^{+} NE forms continuous structure to Lmx1a^{+} NE (**s-u**). (**v**) Schematic of hESC-derived dorsomedial telencephalic tissues which have choroid plexus-, hem-, and medial pallium-like regional patterning, as seen *in vivo*. Scale bars, 500 μm in (**d, g**), 200 μm in (**h, k-u**), 100 μm in (**b, e-f, j**), and 50 μm in (**c**). Nuclear counter-staining (blue), DAPI.

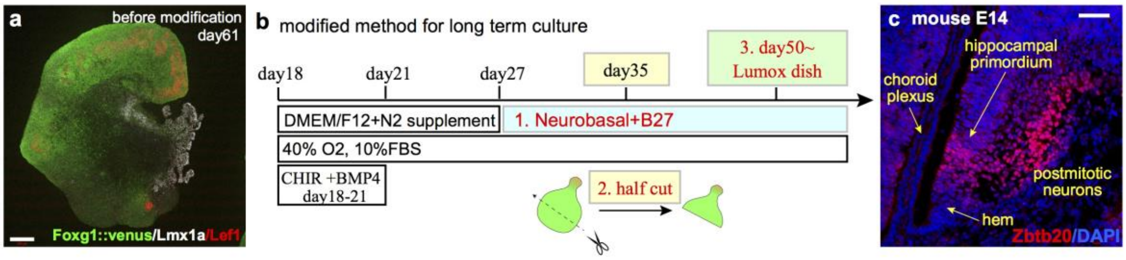

Supplementary Figure 3 | Modified points for long-term culture of hESC-derived medial pallium-like tissues.

(Supplement to Fig. 3)

(**a**) Immunostaining of an aggregate before modification at day61. (**b**) The schematic of modified method for long-term culture of medial pallium-like tissues. The basal media was changed to Neurobasal medium at day 27, the aggregate was cut into half size at day 35 and then transferred into the dishes with high O_2-penetrating bottoms (Lumox dish) at day 50. (**c**) Zbtb20 expression at an early developmental stage in mouse embryo (E13.5-14.5). Zbtb20 expression was observed in postmitotic pyramidal and granule cells, and to a much lesser extent, in the proliferative ventricular zone. Scale bar, 200 μm in (**a**) and 50 μm in (**c**). Nuclear counter-staining (blue), DAPI.

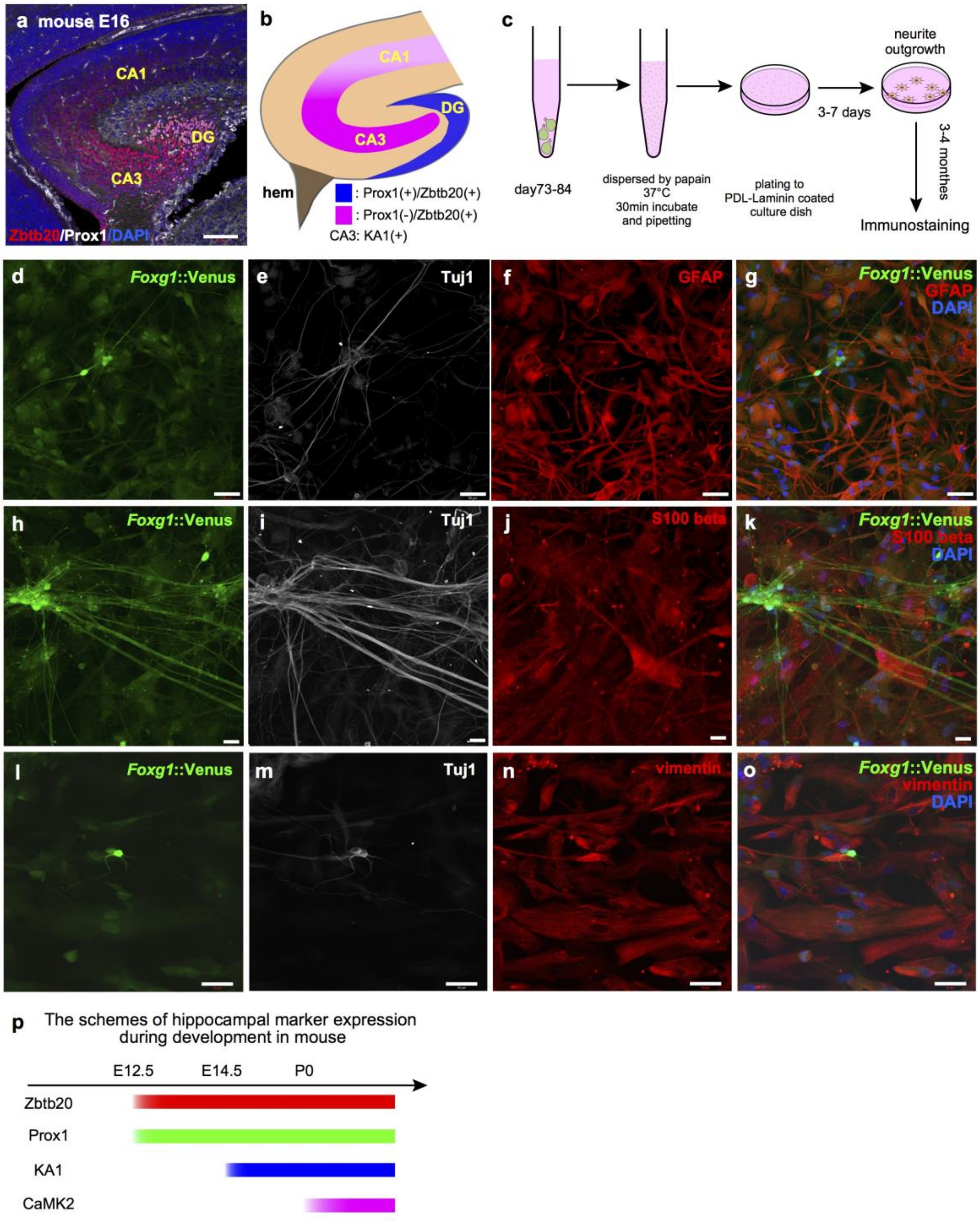

Supplementary Figure 4 | Hippocampal marker expression in mouse, schematic of dissociation culture, and generation of astrocyte-like cells from hESCs-derived dorsomedial telencephalic tissues.

(Supplement to Fig. 4)

(**a**) Coronal section of mouse at E16. Zbtb20 expressed in entire hippocampus, whereas Prox1 location restricted to DG. (**b**) Schematic of hippocampal marker

expression pattern. KA1 is a marker for CA3. (**c**) Schematic of the protocol for dissociation culture. Cells were dissociated at day 73-84 and plated on PDL-laminin coated plates. Dissociated cells attached to the dish within one day, and 3-7 days later, neurite outgrowth was observed. Moreover, *Foxg1*::Venus^{+}/GFAP^{+} glial-like cells were also detected (**d-g**). Other markers for astrocyte such as S100 beta (**h-k**) and vimentin (**l-o**) were also detected in the glial-like cells. (**p**) Schematic of hippocampal marker expression timing in embryonic to postnatal stage in mouse.

Scale bar, 100 μm in (**a**), 50 μm in (**d-g**, **l-o**), and 20 μm in (**h-k**) . Nuclear counter-staining (blue), DAPI.

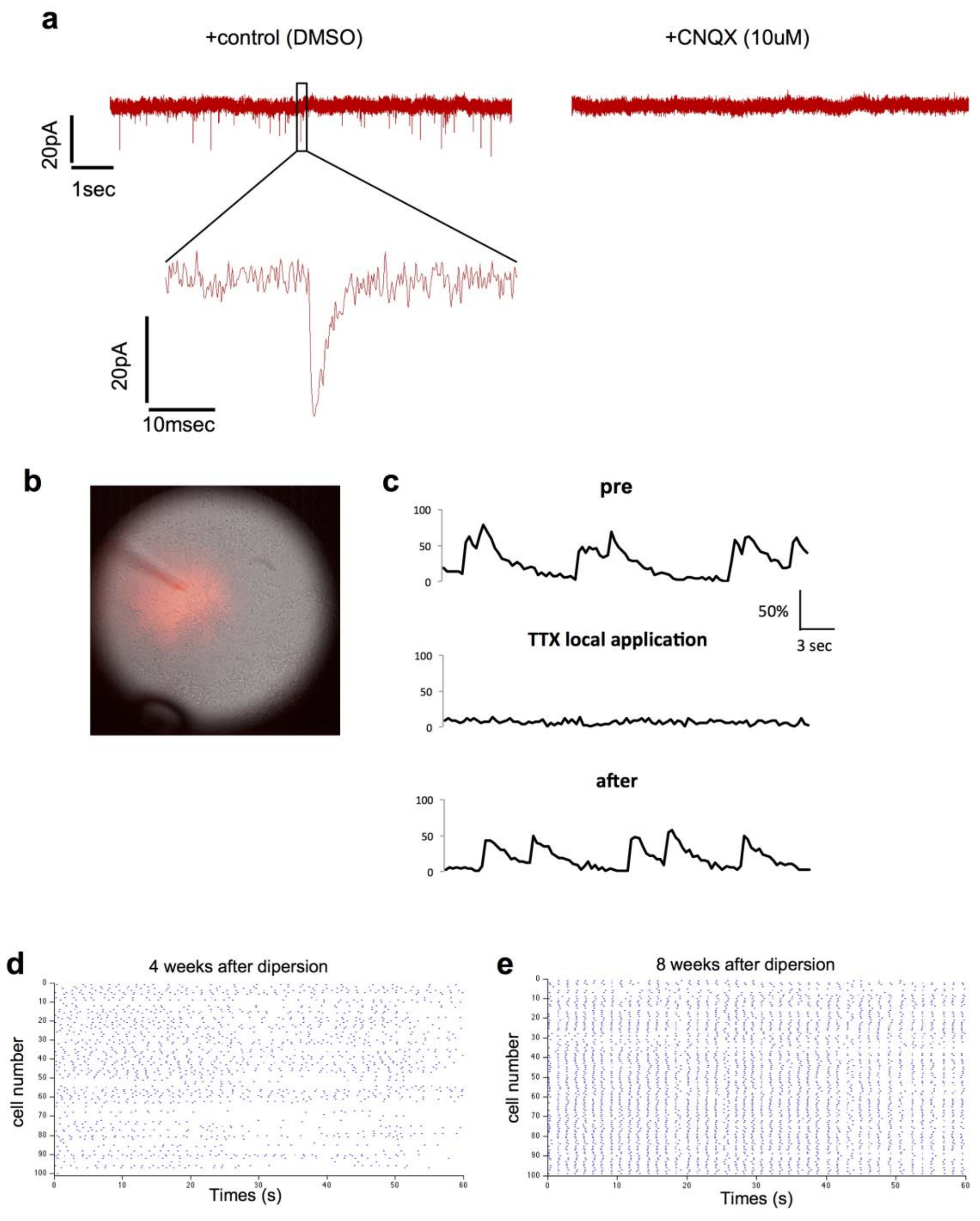

Supplementary Figure 5 | Functional analysis of dissociated hippocampal-like neurons and time-dependent promotion of synchronized oscillation.

(Supplement to Fig. 5)

(**a**) The cultured neurons had spontaneous excitatory postsynaptic currents (EPSCs), and EPSCs were blocked by bath application of CNQX, an antagonist of non-NMDA receptors. (**b-c**) Examination for intracellular calcium dynamics using Fluo4-AM

showed that individual neurons had spontaneous calcium transients. These transients were inhibited by the Na^+ channel blocker tetrodotoxin (TTX) and recovered after ceasing TTX treatment. A representative image of TTX local application (Alexa 647-conjugated dextran) (**b**). Trace images of calcium transient for pre-treatment (upper), TTX local application (middle), and 10 minutes after ceasing TTX application (lower) were shown (**c**). (**d-e**) Raster plot for spontaneous calcium transients of one hundred cells. At 4 weeks after dispersion, calcium transients showed temporally varied activity (**d**). (**e**) At 8 weeks after dispersion, spontaneous transients led to synchronization of network activity.
